# Hydrogen sulfide mediates the protection of dietary restriction against renal senescence in aged F344 rats

**DOI:** 10.1038/srep30292

**Published:** 2016-07-26

**Authors:** Wen-juan Wang, Guang-yan Cai, Yi-chun Ning, Jing Cui, Quan Hong, Xue-yuan Bai, Xiao-meng Xu, Ru Bu, Xue-feng Sun, Xiang-mei Chen

**Affiliations:** 1 Department of Nephrology, Chinese PLA General Hospital, Chinese PLA Institute of Nephrology, State Key Laboratory of Kidney Diseases, National Clinical Research Center for Kidney Diseases, Beijing 100853, China; 2 School of Medicine, Nankai University, TianJin 300071, China

## Abstract

Renal aging is always accompanied by increased oxidative stress. Hydrogen sulfide (H_2_S) can be up-regulated by 50% dietary restriction (DR) for 7-day and can block mitochondrial oxidative stress. H_2_S production exerts a critical role in yeast, worm, and fruit fly models of DR-mediated longevity. In this study, we found that renal aging could be attenuated by 30% DR for 6-month (DR-6M) and life-long (DR-LL), but not for 6-week (DR-6W). The expressions of cystathionine-γ-lyase (CGL) and cystathionine-β- synthase (CBS) were improved by DR-6M and DR-LL. Endogenous H_2_S production shared the same trend with CBS and CGL, while glutathione (GSH) didn’t. When comparing efficiencies of DR for different durations, more evident production of H_2_S was found in DR-6M and DR-LL than in DR-6W. Finally the level of oxidative stress was improved by DR-6M and DR-LL rather than by DR-6W. It concluded that aged rats had the ability to produce enough H_2_S on 30% DR interventions protecting against renal aging, and the effect of DR for long-term were more significant than that of DR for short-term.

Increasing age is an independent risk factor for chronic kidney disease^1^. For the kidney structure, aging is associated with decreased kidney weight, vascular sclerosis, tubular atrophy and interstitial fibrosis; For kidney function, aging not only aggravates the declining processes of the glomerular filtration rate, urinary sodium excretion and erythropoietin production, but also leads to increased glomerular capillary pressure and susceptibility to nephrotoxic injury, including drug-induced renal damage^1^,^2^,^3^. All of these results suggest that delaying or reversing the process of renal aging is necessary to reduce the incidence of age-related kidney dysfunctions and pathological changes.

In 1957, the free radical theory of aging was first proposed. Free radical attack caused macromolecular impairments and accelerated the aging progression, laying the foundation for oxidative stress^4^. In 1990, Sohal *et al*. established that oxidative stress was a causal factor in differentiation and aging^5^. Oxidative stress refers to an imbalance in reduction-oxidation reactions. Once reactive oxygen species (ROS) can’t be effectively cleared, they will produce various cascade reactions that lead to tissue damage and induce or accelerate physiological aging^6^,^7^. NOX2/gp91, as the first identified factor of nicotinamide adenine dinucleotide phosphate oxidase (NOX) which transfers electrons across biological membranes, is responsible for the generation of ROS^8^. ROS avidly reacts with a large number of molecules, including small inorganic molecules as well as lipids, proteins, nucleic acids, and carbohydrates. As a result, it leads to significant increases in malondialdehyde (MDA), protein carbonyl (PC), 8-hydroxygluanine (8-OHdG), and some other oxidation products^8^,^9^. What’s more, oxidative stress also induces apoptosis increase, mitochondrial autophagy decrease, erythropoietin production restriction, and sodium homeostasis disorder in kidneys^10^,^11^. All the data above show that oxidative stress participates in the process of renal aging and age-related alternations directly and indirectly.

Currently, it is widely accepted that DR, as a natural regimen of non-genetic transformation without malnutrition, brings numerous and beneficial effects, especially in extending the maximum and mean lifespan of a variety of organisms, from yeast to humans^12^. DR encompasses various forms, including the reduction of 30% to 50% of total calorie, protein and even essential amino acid intake for short-term or for life-long^11^,^13^. Specifically restriction of essential amino acids (EAAs), especially Met, controls the benefits of longevity in diverse organisms, such as yeast, files, worms, elegans and rodents^14^,^15^,^16^,^17^. Lifespan is reduced by the addition back of some amino acids, in particular sulfur amino acid (SAA), indicating that restriction of SAA takes on a common role to mediate numerous benefits of DR^15^. Recently most of the biological mechanisms that underlie DR are focused on nutrient-sensing pathway activity, including the NAD^+^/sirtuins pathway^18^, adenosine monophosphate-activated protein kinase^19^, mammalian target of rapamycin^20^, and insulin-like growth factor^21^. Similarly, SAA restriction extends lifespan in many species not only by altering insulin-like growth factor I, glucose, and insulin levels, but also by increasing the level of macrophage migration inhibition factor and the capacity of stress resistance^16^. Metformin, as an indispensable molecule regulating energy metabolism, retards aging in C. elegans mainly by altering methionine metabolism and microbial folate, indicating that transsulfuration pathway (TSP) has an essential role in the protection of metformin against aging^17^. TSP is responsible for methionine metabolism to produce endogenous H_2_S. Zhang *et al*. reviewed that H_2_S delayed the progression of aging mainly by inhibiting oxidative stress, activating silent information regulator of transcription 1 (SIRT1), and probably suppressing the expression of klotho^22^. Recently, Hine *et al*. found that TSP and H_2_S mediated the beneficial effects of DR in the hepatic and renal ischemia-reperfusion models, and they also speculated that H_2_S could participate in delaying aging^23^. Based on these data, it comes to the conclusion that increased TSP activity is an evolutionarily conserved effector to multiple DR regimens, including protein restriction, caloric restriction, and SAA restriction, and that increased H_2_S production under these conditions indicates a common molecular mechanism underlying multiple DR benefits.

H_2_S has been gaining increasing attention as a third signal gasotransmitter, following nitric oxide and carbon monoxide. It exerts beneficial effects mainly through antioxidant activity at physiological levels^12^,^24^. It has been reported that H_2_S and GSH are endogenously generated by CGL and CBS in TSP^23^,^25^,^26^. The expressions of CBS and CGL increase when Cys is low to allow de novo synthesis from Met (Fig. 1)^27^. H_2_S influences the normal physiology of kidney as well as the pathogenesis of kidney diseases^25^,^28^. It has been suggested that the elevated H_2_S concentration in kidneys not only mediates the metabolism of homocysteine, but also regulates vascular and tubular functions, along with increased renal blood flow, glomerular filtration rate and urinary excretion^26^. Because of its diverse properties and systemic effects, H_2_S dysfunction has recently been identified as a key factor in the onset and progression of renal diseases, such as ureteral obstruction^29^, chronic kidney disease^30^, drug-induced nephrotoxicity^31^ and renal ischemia reperfusion injury^32^. Existing evidences show that H_2_S is involved in delaying the progression of aging and age-associated diseases by inhibiting oxidative stress, activating SIRT1, and probably exerting indirect effects on anti-aging gene klotho^22^. Therefore, it would be interesting to investigate whether aged rats have the ability of producing sufficient H_2_S on the intervention of DR for different durations to delay the process of aging.

This study, for the first time, observed whether aged rats had the ability to produce enough H_2_S on the intervention of 30% DR in delaying kidney aging, and compared the efficiencies of DR for different durations (6-week, 6-month, and life-long) on the production of H_2_S. Firstly, we observed that metabolic indexes, renal function, renal histology alternations and senescence markers on 30% DR for different durations. Secondly, we systematically detected the changes of both expressions and localizations of CBS and CGL in TSP and measured the levels of H_2_S and GSH. Finally we observed the levels of ROS, oxidation products and anti-oxidative indicators. We found that aged rats had the ability to produce enough H_2_S, but not GSH, to delay the process of aging, and it’s more significant in DR-6M and DR-LL groups than in DR-6W group. DR-induced H_2_S production would have an ROS-scavenging ability and antioxidant-improving properties to attenuate stress-induced senescence in the aged kidneys to some extent.

## Results

### Animal characteristics

Compared with young ad libitum (Young-AL), the body weight, serum urea nitrogen, the urine protein/urine creatinine ratio, triglycerides, and serum glucose in old ad libitum (Old-AL) were significantly increased (see Supplementary Table S1, p < 0.05), while the kidney weight/body weight ratio in Old-AL was decreased (see Supplementary Table S1, p < 0.05). There was no change in the levels of serum creatinine, serum cholesterol, total protein and albumin in two groups. Compared with corresponding AL control group, significant improvements in the levels of body weight, kidney: body weight, serum urea nitrogen, urine protein/urine creatinine ratio, triglycerides, serum glucose were found in both DR-6M and DR-LL (see Supplementary Table S2, p < 0.05), but there was no obvious difference in other indexes (see Supplementary Table S2, p > 0.05). While it showed no obvious changes of all the indexes in DR-6W group (see Supplementary Table S2, p > 0.05). Compared with the effect of DR-6W in improving kidney weight/body weight and triglycerides, it was more prominent in DR-LL than in DR-6M (see Supplementary Table S2, p < 0.05).

### Kidney structural alterations

Renal tissues were processed by routine Periodic Acid-Schiff (PAS) staining. Specific morphological changes and pathological grading were shown in Supplementary Figs S1 and S2. Glomerular lesions and interstitial renal tubular damage, such as glomerulosclerosis, fibrous, cell proliferation, renal tubule atrophy, renal tubular epithelial cell degeneration, renal tubular casts and inflammatory cell infiltrations, significantly increased in Old-AL (see Supplementary Fig. S1, p < 0.05). Compared with AL corresponding, renal tubular epithelial cell degeneration and glomerulosclerosis were improved in both DR-6M and DR-LL (see Supplementary Fig. S2, p < 0.05) rather than DR-6W (see Supplementary Fig. S2, p > 0.05). Some interstitial fibrosis, cell proliferation, loop necrosis and inflammatory cell infiltrations were largely blunted largely by DR-LL and partly by DR-6M (see Supplementary Fig. S2, p < 0.05), but there was little change in DR-6W (see Supplementary Fig. S2, p > 0.05).

### Senescence markers in aged rat renal tissues

**Figure 2:**
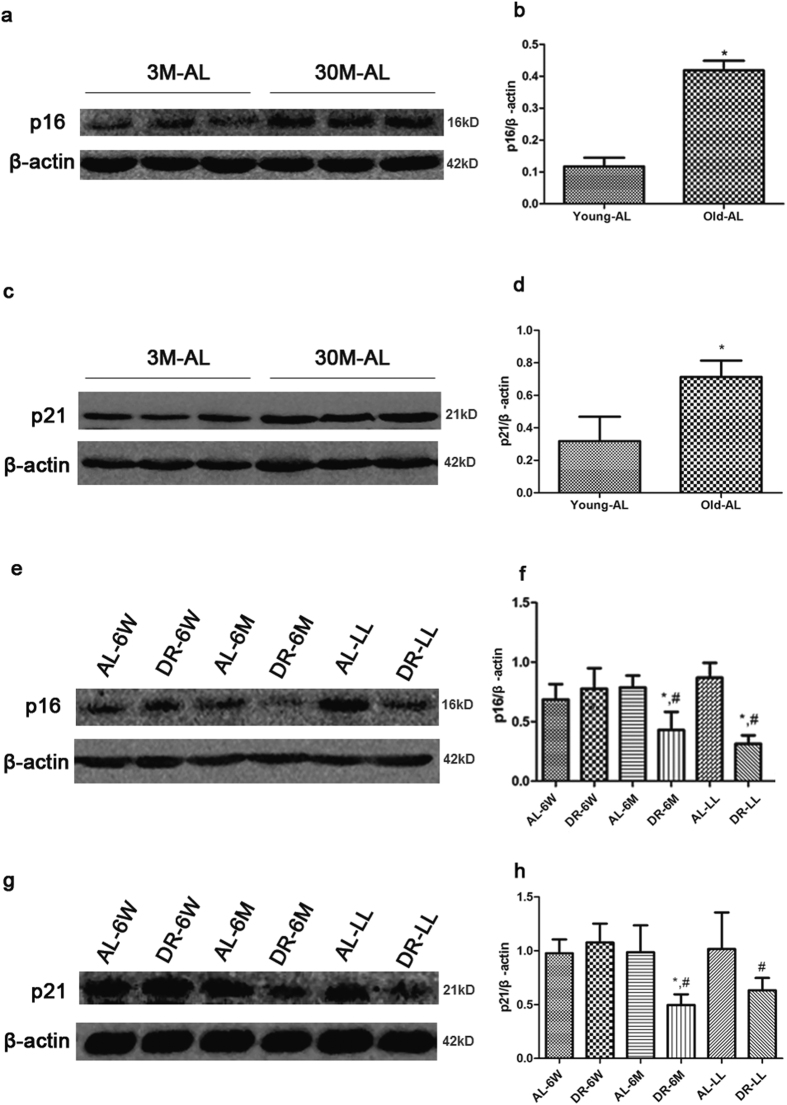
The expressions of senescent biomarker p16 and cell cycle inhibitor p21 in kidneys. Western blot results (**a,c**) and quantitative analysis of the band density (**b,d**) showed that the expressions of p16 and p21 were increased in kidneys of Old-AL vs. those of Young-AL. Western blot results (**e,g**) and quantitative analysis of the band density (**f,h**) showed that DR-6M and DR-LL decrease the expressions of p16 and p21 but DR-6W doesn’t. The data are presented as the mean ± SD (n = 5–8). *p < 0.05 vs. the corresponding AL. ^#^p < 0.05 vs. DR-6W.

In this study, both the expression of p16, as a possible effector and a robust biomarker in mammalian aging, and the expression of p21, as a cell cycle inhibitor and the most extensive kinase inhibitor, were higher in Old-AL than in Young-AL (Fig. 2a–d, p < 0.05). To observe the effects of DR for different durations on aged kidney, the expressions of p16 and p21 in aged rats from different groups were further detected. As shown in Fig. 2, the expression of p16 protein could be reversed by both DR-6M and DR-LL, but not by DR-6W (Fig. 2e,f, p < 0.05). Similarly cell cycle inhibitor p21 showed the same trend (Fig. 2g,h, p < 0.05).

### Alternations of capital enzymes, H_2_S, and GSH in TSP

**Figure 3:**
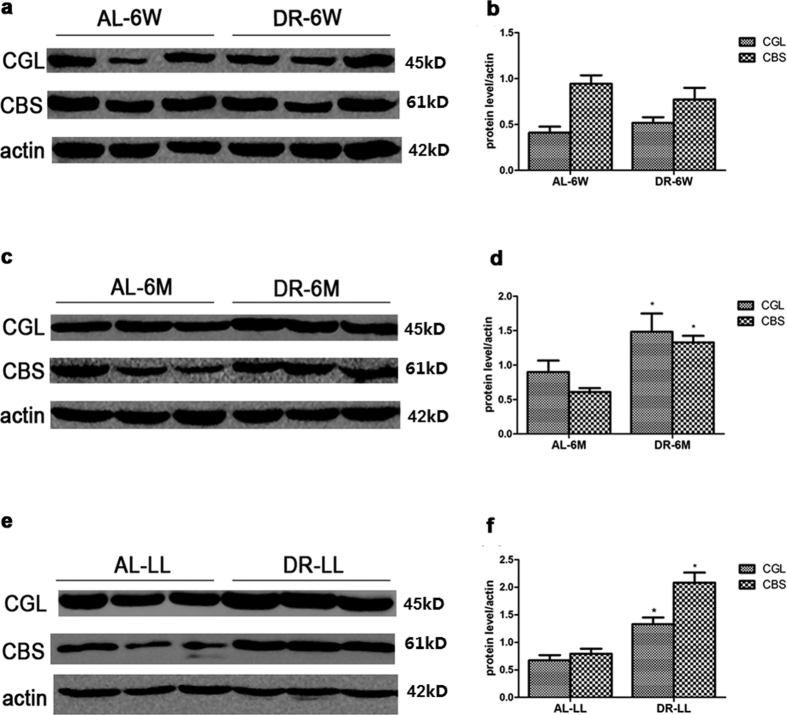
Expressions of CBS and CGL in every group. Western blot results of CBS and CGL expression in (**a**) AL-6W and DR-6W, (**c**) AL-6 M and DR-6M, (**e**) AL-LL and DR-LL. (**b,d,f**) Quantitative analysis of the band density for CBS and CGL. The data are presented as the mean ± SD (n = 5–8). *p < 0.05 vs. the corresponding AL.

**Figure 4:**
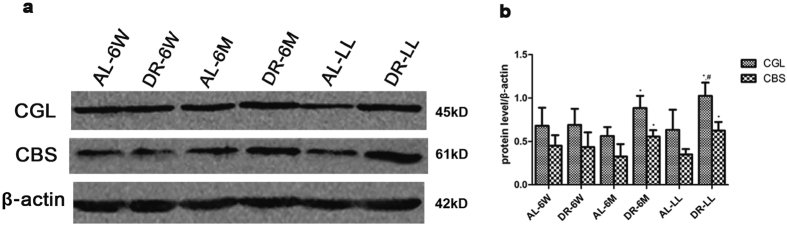
Comparisons of CGL and CBS in kidneys were attenuated by DR for different durations. (**a**) Western blot results and (**b**) quantitative analysis of CBS and CGL in every group. DR-6M and DR-LL, rather than DR-6W, significantly enhanced the expressions of CGL and CBS. The data are presented as the mean ± SD (n = 5–8). *p < 0.05 vs. the corresponding AL. ^#^p < 0.05 vs. DR-6W.

**Figure 5:**
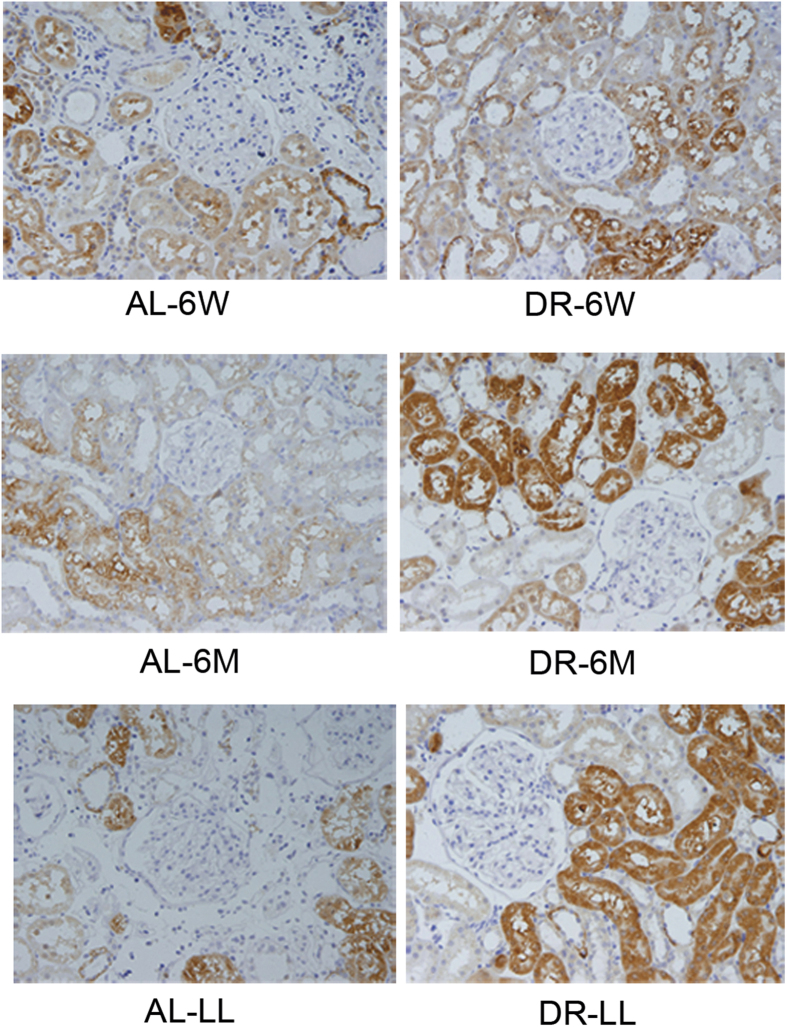
Location of CGL in renal tissue by immunohistochemistry staining. Magnification, x400. It was mainly expressed in the renal tubular cytoplasm, but rarely in glomerulus.

**Figure 6:**
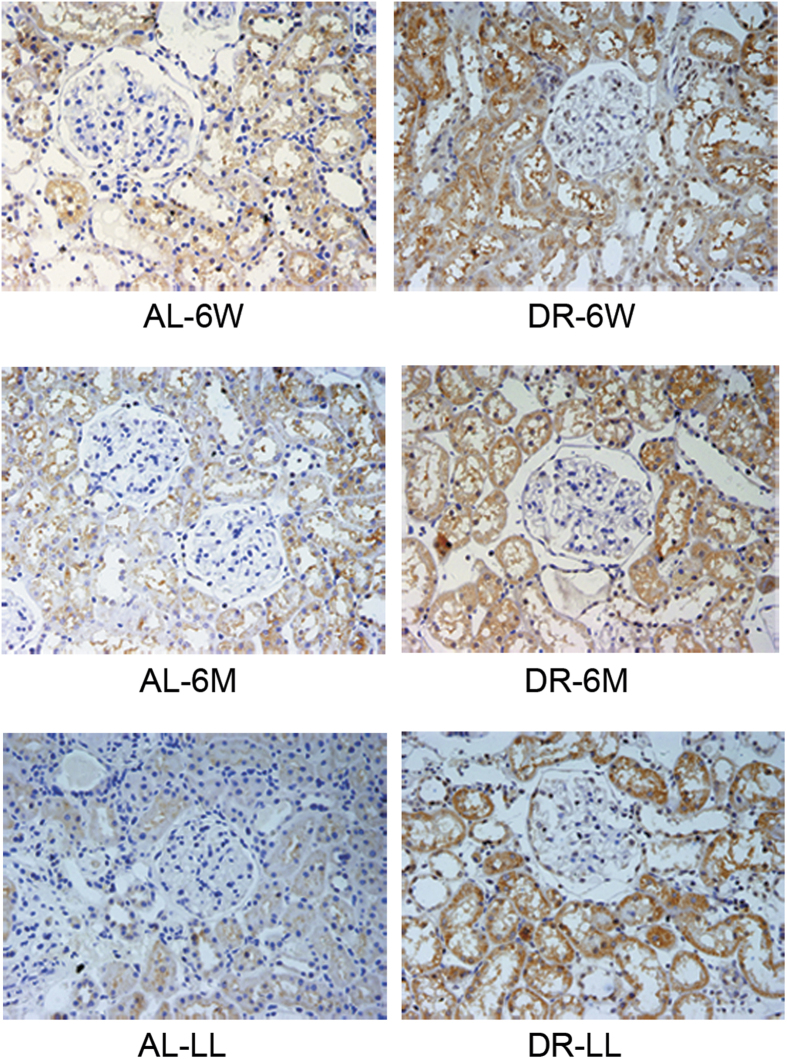
Location of CBS in renal tissue by immunohistochemistry staining. Magnification, x400. It was mainly expressed in the renal tubular cytoplasm.

Firstly we observed the expressions of the capital enzymes in the TSP. There was little difference in the expressions of both CGL and CBS in DR-6W (Fig. 3a,b, p > 0.05). It also showed that there were increases in both CGL and CBS in DR-6M (Fig. 3c,d, p < 0.05) and DR-LL (Fig. 3e,f, p < 0.05). We further observed the trends in the endogenous H_2_S-related enzymes in groups of DR for different durations together. Compared with the corresponding AL groups, the levels of H_2_S-related enzymes were respectively improved by DR-6M and DR-LL rather than by DR-6W (Fig. 4a,b, p < 0.05); Compared with DR-6W, it took on a more significant improvement in DR-LL than DR-6M, which demonstrated that the expressions of the TSP enzymes were increased in a time-dependent mode to some extent (Fig. 4a,b, p < 0.05). We also determined the expressions of CGL and CBS in aged kidneys with an immunohistochemistry stain, which showed the remarkable alterations and locations of these two enzymes (Figs 5 and 6). Consistent with previous findings, both CGL and CBS were primarily expressed in renal tubular epithelial cells, whereas they were hardly expressed in the glomerulus.

**Figure 7:**
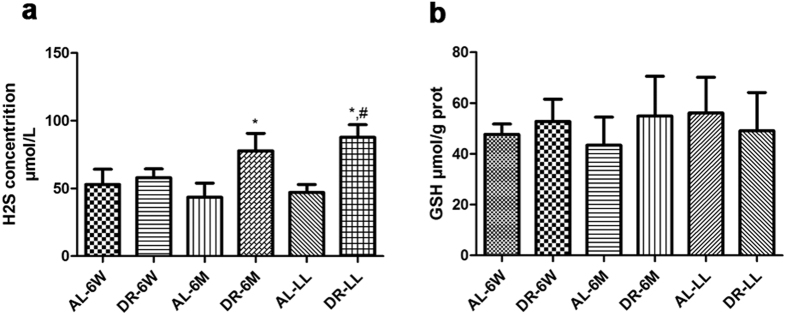
Effects of DR for different durations on H_2_S and GSH level. It could be improved by DR-6M and DR-LL in the levels of H_2_S, rather than GSH, in kidney tissues. The data are presented as the mean ± SD (n = 5–8). *p < 0.05 vs. the corresponding AL. ^#^p < 0.05 vs. DR-6W.

We applied enzyme-linked immunosorbnent assay to quantify H_2_S and GSH in aged kidney tissues and found that the expression of H_2_S was improved by DR-6M and DR-LL (Fig. 7a, p < 0.05) while the expression of GSH was not (Fig. 7b, p > 0.05). Compared with DR-6W, the effect of DR-LL on H_2_S was more substantial than that of DR-6M (Fig. 7a, p < 0.05).

### Oxidant properties and antioxidant capacity

**Figure 8:**
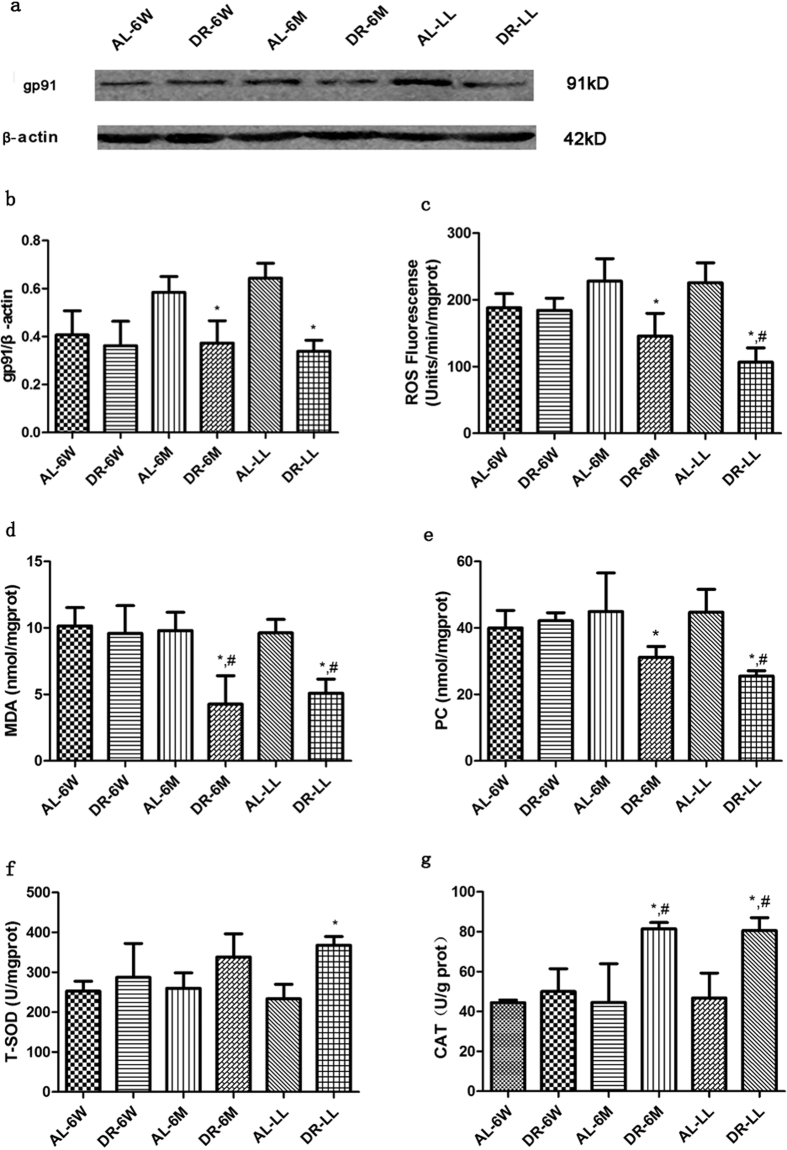
DR for different durations improved the renal oxidative stress. (**a**) The expressions and (**b**) the quantitative analysis of the band density of gp91, and (**c**) the levels of ROS increased with aging, which could be reduced by DR-6M and DR-LL in the kidney tissues. The biochemical measurements with regard to the oxidative stress in aged kidney tissues, such as (**d**) CAT, (**e**) PC, (**f**) T-SOD, and (**g**) CAT, were improved with the interventions of DR-6M and DR-LL, rather than of DR-6W. The data are presented as the mean ± SD (n = 5–8). *p < 0.05 vs. the corresponding AL. ^#^p < 0.05 vs. DR-6W.

Western blot analysis showed that the expressions of NOX2/gp91 were significantly reduced in DR-6M and DR-LL (Fig. 8a,b, p < 0.05), and the production of ROS showed a similar trend as that of gp91/NOX2 (Fig. 8c, p < 0.05). Then we detected oxidant properties (MDA, and PC) and anti-oxidant indicators (catalase, CAT and total superoxide dismutase, T-SOD) in aged kidneys. It showed that the levels of MDA and PC had been reduced by DR-6M and DR-LL (Fig. 8d,e, p < 0.05) rather than by DR-6W (Fig. 8d,e, p > 0.05). Compared with DR-6W, it showed a significant reduction in expressions of both MDA and PC in DR-LL (Fig. 8d,e, p < 0.05), while it showed a vital decrease in expression of only MDA in DR-6M (Fig. 8d, p < 0.05). Finally we observed the expressions of T-SOD and CAT. Compared with the corresponding AL, the level of T-SOD was improved only by DR-LL (Fig. 8f, p < 0.05), It was enhanced in the expression of CAT in both DR-6M and DR-LL, but not in DR-6W (Fig. 8g, p < 0.05).

In addition, we also examined the effects of DR for different durations on the products of oxidative DNA damage in old renal tissues. According to the immunochemical staining results of 8-OHdG, we observed that it was mainly distributed in renal tubules (see Supplementary Fig. S3). Then we compared the effects of DR for different durations on renal 8-OHdG levels, and found that the immunoreactivities and the staining intensities of 8-OHdG in DR-6M and DR-LL groups were significantly lower than the corresponding AL groups, indicating that DR for long term could suppress the oxidative injury in old kidneys (see Supplementary Fig. S3, p < 0.05).

In short, DR-6M and DR-LL enhanced the expressions of CGL and CBS, contributing to elevating the levels of H_2_S rather than GSH. Thus, our study demonstrated that aged rats had the ability of producing enough H_2_S and that it was more significant in DR for long-term than in DR for short-term, which indicating that TSP and H_2_S could mediate the DR effect in protecting against renal senescence partially by modulation of redox balance to some extent.

## Discussion

Accumulated studies have shown that aging is an independent risk factor for the onset and development of renal diseases^33^,^34^. DR can extend the lifespan or delay the aging process by decreasing oxidative stress level^35^,^36^,^37^. However, the specific mechanisms among DR, oxidative stress and aging remain unclear. Recently there was an study highlighted that 50% DR for 7 days could partly mediate the protection of DR and further increased expressions of CGL and CBS, resulting in the production of H_2_S in young mice model^23^. In this study, we attempted to explore whether the aged rats had the potentiality to produce enough H_2_S, and further to observe the efficiencies of DR for different durations in delaying renal aging.

To the best of our knowledge, this study was the first time to study protection against renal senescence by DR for different durations. We observed metabolic indexes, renal function, renal histology alternations and senescence markers, and found that DR-6M and DR-LL could significantly delay or reduce multiple abnormal or pathological manifestations of aged kidneys. We further respectively measured the levels of capital enzymes, H_2_S, GSH, and oxidative stress. And we found that it was H_2_S, not GSH, that worked in the protection against stress-induced senescence in aged kidneys by reducing oxidative stress. Then we compared the efficiencies of H_2_S induced by DR for different durations, and concluded that the effect of DR for long-term was better than that of DR for short-term.

It has been widely accepted that oxidative stress activity increases during organism aging and that the imbalance between oxidant substances and antioxidant products exerts a significant and causal factor in age-associated symptoms^4^,^5^,^38^. Growing evidences indicate that a progressive accumulation of oxidative stress is involved in lipid, protein, and DNA damage, disturbing physiological homeostasis and contributing to aging-related kidney dysfunctions^7^,^39^. A previous study in our laboratory confirmed that 40% DR for 8 weeks reduced the expression of 8-OHdG, a sensitive biomarker for mitochondrial DNA (mtDNA), and thus protected the kidney from oxidative damage in aged rats^35^,^37^. The above results indicate that the increased oxidative stress in aged kidney can be decreased by DR.

H_2_S, which is previously regarded as a poisonous gas, has been gaining increasing recognition for its numerous beneficial effects. Recently, a study demonstrated that the expressions of both H_2_S and H_2_S-related enzymes increased with 50% DR for 7 days or methionine restriction, and further blocked mitochondrial oxidative stress^23^. It is well known that TSP is responsible for the production of both H_2_S and GSH^23^. In our study, TSP was evidently activated, and it was H_2_S, rather than GSH, that increased in DR-6M and DR-LL groups. Compared with DR-6W, DR-LL exerted larger roles in H_2_S production than DR-6M, indicating it was necessary to maintain a long-term and regular rhythm of dietary restrictions.

Accumulated studies have shown that the role of H_2_S has been widely considered as a direct and/or indirect mediator of renal oxidative stress response^22^,^28^. H_2_S exerts protection from stress in part by inner membrane component sulfide quinone oxidoreductase and the latter transfers electrons from H_2_S to the electron transport chain and coenzyme Q^40^,^41^. Moreover, thiosulfate, as a product of H_2_S oxidation via sulfide quinone oxidoreductase, can encounter further chemical modification in a thioredoxin-reductase-dependent reaction using glutathione to produce sulfate or sulfite, which serves as terminal electron acceptors for ATP production and thus results in H_2_S generation in some unicellular organisms^41^,^42^. Wu *et al*. noted that exogenous NaHS (100 μmol/L) treatment decreased ROS production and enhanced SOD, GSH and GST expression in H9c2 cardiomyocytes, while Ex 527 (10 μmol/L) reversed these protection effects significantly, which suggests that SIRT1 participated in anti-oxidative activities of H2S during cellular senescence^43^,^44^. Consistent with previous research, our study found that H_2_S was involved in inhibiting free-radical generation, such as MDA PC, and 8-OHdG. One study suggested that the anti-oxidant actions of H_2_S were mediated by preventing p66Shc phosphorylation, thus inhibiting mitochondrial ROS production^45^. Obviously, there are other mechanisms that involves in suppressing cellular senescence besides the sulfhydration of Kelch-like ECH-associated protein 1 and 8-nitroguanosine-3′, 5′-cyclic monophosphate^46^,^47^,^48^. Many studies have concluded that H_2_S protected kidney aging mainly by improving the level of oxidative stress, while specific molecular mechanisms remain to be validated by future investigations.

In summary, we demonstrated for the first time that aged rats had the ability to produce enough H_2_S on the interventions of DR-6M and DR-LL, thus decreasing stress-induced senescence in aged kidneys. It was implied that H_2_S could be a unified mediator of DR in reducing age-related oxidative stress. These novel results could be meaningful for identifying the increasingly important and complex roles of the H_2_S system in stress-induced aging, which indicated that H_2_S could be likely to be applied in improving the clinical outcomes of aging-related renal diseases in the future.

## Methods

### Animals

This study was approved by the Chinese PLA General Hospital and Military Medical Postgraduate College. It was performed in accordance with the National Institutes of Health guidelines for the use of experimental animals. The rats were approved in a 12:12 light/dark cycle at 22 ± 1 °C and 50 ± 10% relative humidity. They were fed with one male per cage and had free access to water under a specific pathogen-free condition. Young (3 months, n = 48) male Fischer 344 rats were divided into ad libitum dietary (AL) group (Young-AL, n = 40) and dietary restriction (DR) group (n = 8). The rats in the DR groups were fed with food that was approximately 70% of the food consumed by the AL group. The food consumption in AL group was measured every week, and then, the results obtained were used to calculate the daily food intake for the next week. When the rats were 24 months old (Old-AL), the AL groups were further divided into AL (n = 24) and DR (n = 16) groups, and then, 8 rats out of the AL and DR groups were sacrificed after 6-week DR (AL-6W and DR-6W, respectively). When they were 30 months old, all of the rats in each group (AL-6M, DR-6M, AL-LL and DR-LL) were sacrificed. Twenty-four hour urine samples were collected in metabolic cages individually and then were sent to detect the urinary protein/creatinine ratio by the coomassie brilliant blue method and the sarcosine oxidase method. Blood samples were collected from the inner canthus before the animals were sacrificed and analyzed. Specially, triglyceride and blood urea nitrogen were detected by colorimetric methods. The hexokinase method was used to detect blood sugar and enzymatic methods were used to detect total cholesterol and serum creatinine. The kidneys were stored at −80 °C for immunohistochemistry staining and western blot.

### Renal histology and histological grading

Kidney specimens were fixed in a 10% formalin solution at 4 °C overnight. After a series of graded alcohol dehydration, kidney pieces were embedded, sectioned and stained with PAS. The images were visualized and captured at a total magnification of ×200 with Nikon Element software (Nikon Instrument, Nikon Inc., Melville, NY, USA) and were analyzed by two investigators according to the standards of glomerular lesions and tubulointerstitial lesions previously described^49^,^50^. Each specimen was measured from 20 random fields per rat using the National Institutes of Health (NIH, MD, USA) Semi-Quantitative Score. In particular, glomerular Lesions (inflammatory cell infiltration, glomerulosclerosis, fibrous and cellular crescents, loop necrosis/karyorrhexis) and renal interstitial lesion (protein casts) were graded by standard scores of 0 points (0%), 1 point (<25%), 2 points (25–50%), and 3 points (>50%). Furthermore, renal interstitial lesions (renal tubular epithelial cell degeneration, renal tubular atrophy, renal interstitial fibrosis and interstitial inflammatory cell infiltration) and glomerular lesions (cell proliferation) were measured by standard scores of 0 points (−), 1 point (mild), 2 points (medium), and 3 points (severe).

### Western blot analysis

After the kidney tissues were lysed in radio immunoprecipitation assay buffer, the extracted protein concentration was measured with a BCA Protein Assay kit (Thermo Fisher Scientific, Rockford, IL, USA). The extracted proteins were mixed well with 5% sodium dodecyl sulfate-polyacrylamide gel electrophoresis (SEMS-PAGE) sample buffer followed by heating at 95 °C for 10 min. A total of 50 to 80 μg protein was separated using 8–12% SEMS-polyacrylamide gel electrophoresis, transferred to a nitrocellulose (NC) membrane and then inoculated at 4 °C overnight with the primary antibodies: rabbit monoclonal anti-p16 antibody (1:500; Abcam, Cambridge MA, USA), rabbit polyclonal anti-p21 antibody (1:1000; Proteintech, Chicago IL, USA), rabbit polyclonal anti-CGL and anti-CBS antibody (1:1000; Abcam, Cambridge MA, USA), and rabbit monoclonal anti-gp91/NOX2 antibody (1:2000; Abcam, Cambridge MA, USA). The HRP-conjugated secondary antibodies (Santa cruz biotechnology, CA, USA) were incubated at room temperature for 2 h or at 4 °C for 4 h. Each membrane was detected by enhanced chemiluminescence, and densitometry was conducted using Quantity One software (Bio-Rad Laboratories, Hercules, CA, USA).

### Oxidative stress-related indicator detection

The renal tissue was homogenized in a cold 0.9% sodium chloride solution to make a 10% homogenate. Then, the homogenate was centrifuged at 4000 rpm 4 °C for 15 min. The tissue supernatant that was extracted was collected for further analysis. The protein concentration was determined with a BCA Protein Assay kit (Thermo Fisher Scientific, Rockford, IL, USA). The activities of ROS, MDA, PC, CAT, T-SOD and GSH,contents were assayed according to the recommended procedures provided by commercial reagent kits (Jiancheng Institute of Bioengineering, Nanjing, China). The level of H_2_S in kidney was determined in tissue lysates using the Rat Hydrogen Sulfide Assay kit (Neobiolab, Cambridge MA, USA) according to the manufacturer’s instructions.

### Immunohistochemistry staining

The immunohistochemistry staining methods were performed as previously described^35^. After dewaxing, antigen retrieval and blocking of endogenous peroxidase, the sections were fixed with goat serum and incubated with a 1:1000 dilution of rabbit monoclonal anti-CGL antibody (Abcam, Cambridge MA, USA), a 1:2000 dilution of rabbit monoclonal anti-CBS antibody (Abcam, Cambridge MA, USA), and a 1:20 dilution of mouse monoclonal anti-8-OHdG antibody (Santa cruz biotechnology, CA, USA) overnight at 4 °C, followed by incubation with biotin-conjugated goat anti-rabbit IgG (Invitrogen, Carlsbad, CA, USA) and streptavidin- conjugated peroxidase (Invitrogen, Carlsbad, CA, USA). Finally, sections were detected under a microscope after reaction using 3,3′-diaminobenzidine (DAB) (Invitrogen, Carlsbad, CA, USA). The intensity of immunohistochemical staining of 8-OHdG was analyzed in ten random fields (×400) of renal cortex per rat using Image J software (NIH, MD, USA).

### Statistical analysis

All of the data analyses were performed using SPSS 17.0 (SPSS, Chicago, IL, USA) software. For the animal and kidney characteristics as well as the protein expressions, the data were provided as the mean ± SD. Two group differences were determined by T test and multiple group differences were determined by ANOVA analysis.

## Additional Information

**How to cite this article**: Wang, W.-J. *et al*. Hydrogen sulfide mediates the protection of dietary restriction against renal senescence in aged F344 rats. *Sci. Rep*. **6**, 30292; doi: 10.1038/srep30292 (2016).

## Supplementary Material

Supplementary Information

## Figures and Tables

**Figure 1:**
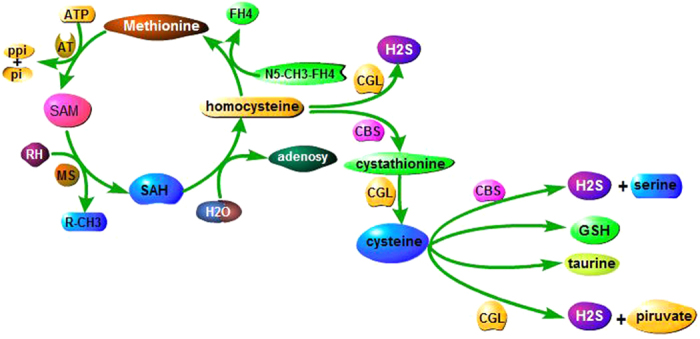
Pathway of the Krebs-Hanseleit cycle and TSP, which were responsible for productions of H_2_S, GSH and some other substances mainly via CBS and CGL. When cystathionine is in the state of lack, it will cause a compensatory enhancement of CGL and CBS in TSP, as a result of increased expression of H_2_S. SAM, S-adenosylmethionine; SAH, S-adenosylhomocysteine; CBS, cystathionine beta-synthase; CGL, cystathionine gamma-lyase; TSP, transsulfuration pathway; H_2_S, hydrogen sulfide; GSH, glutathione.
