# Supplementary material for: Hydrogen sulfide mediates the protection of dietary restriction against renal senescence in aged F344 rats
Source: Sci Rep. 2016 Jul 26;6:30292. doi: 10.1038/srep30292 (PMC4960595; doi:10.1038/srep30292)
Supplement: Supplementary Information [file srep30292-s1.pdf]

Title page

Title: Hydrogen sulfide mediates the protection of dietary restriction against renal senescence in aged F344 rats

The authors' names: Wenjuan Wang, [1,2] Guangyan Cai,[1★] Yichun Ning,[1,2] Jing Cui,[1] Quan Hong,[1] Xueyuan Bai,[1] Xiaomeng Xu,[1,2] Ru Bu,[1] Xuefeng Sun[1] and Xiangmei Chen[1]

The institute: 1. Department of Nephrology, Chinese PLA General Hospital, Chinese PLA Institute of Nephrology, State Key Laboratory of Kidney Diseases, National Clinical Research Center for Kidney Diseases, Beijing 100853, China. 2. School of Medicine, Nankai University, TianJin 300071, China.

Correspondence: Dr. Guangyan Cai[★], Department of Nephrology, Chinese PLA General Hospital, Chinese PLA Institute of Nephrology, State Key Laboratory of Kidney Diseases, National Clinical Research Center for Kidney Diseases, 28, Fuxing Road, HaiDian District, Beijing 100853, China.

Telephone: 86-10-66935462; Fax: 86-10-6813 0297; E-mail: caiguangyan@sina.com

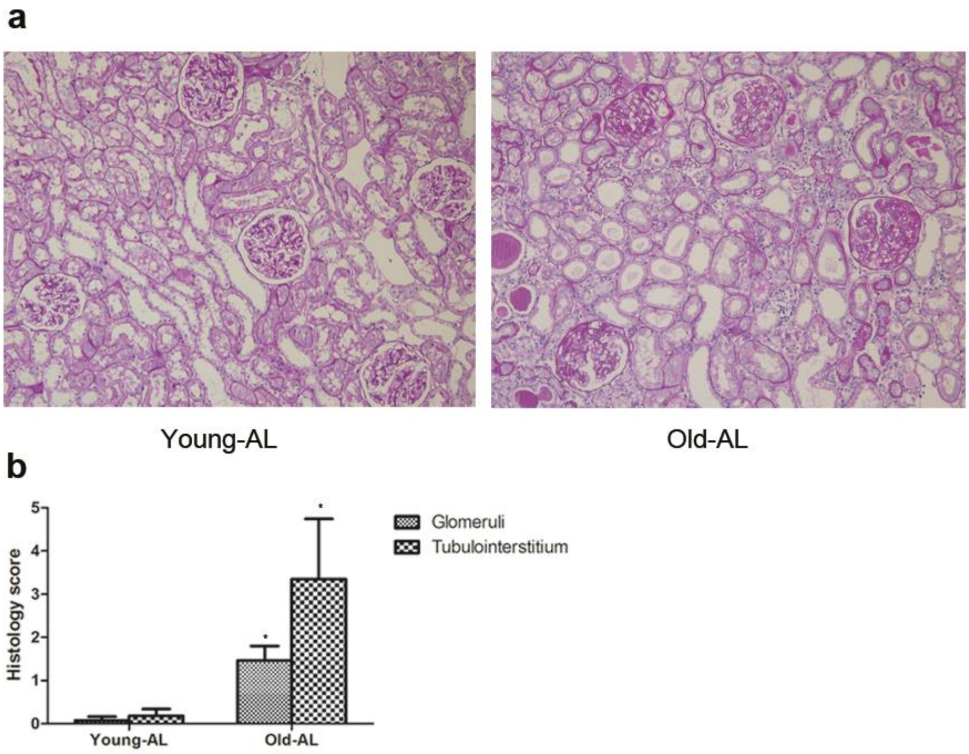

Fig.S1 Photomicrographs and histology scores of renal cortex in young and old rats. a For the Young-AL group, no abnormalities were found, while glomerular and tubulointerstitial lesions were severe in the Old-AL group. Renal tissue sections were stained using PAS staining. Magnification, ×200. b Compared with the Young-AL group, histology scores were significantly increased in the Old-AL group. Renal pathological grading by standard procedures from 20 random fields per rat. The data are presented as the mean ± SD (n = 5-8). *p<0.05 vs. the Young-AL group.

a

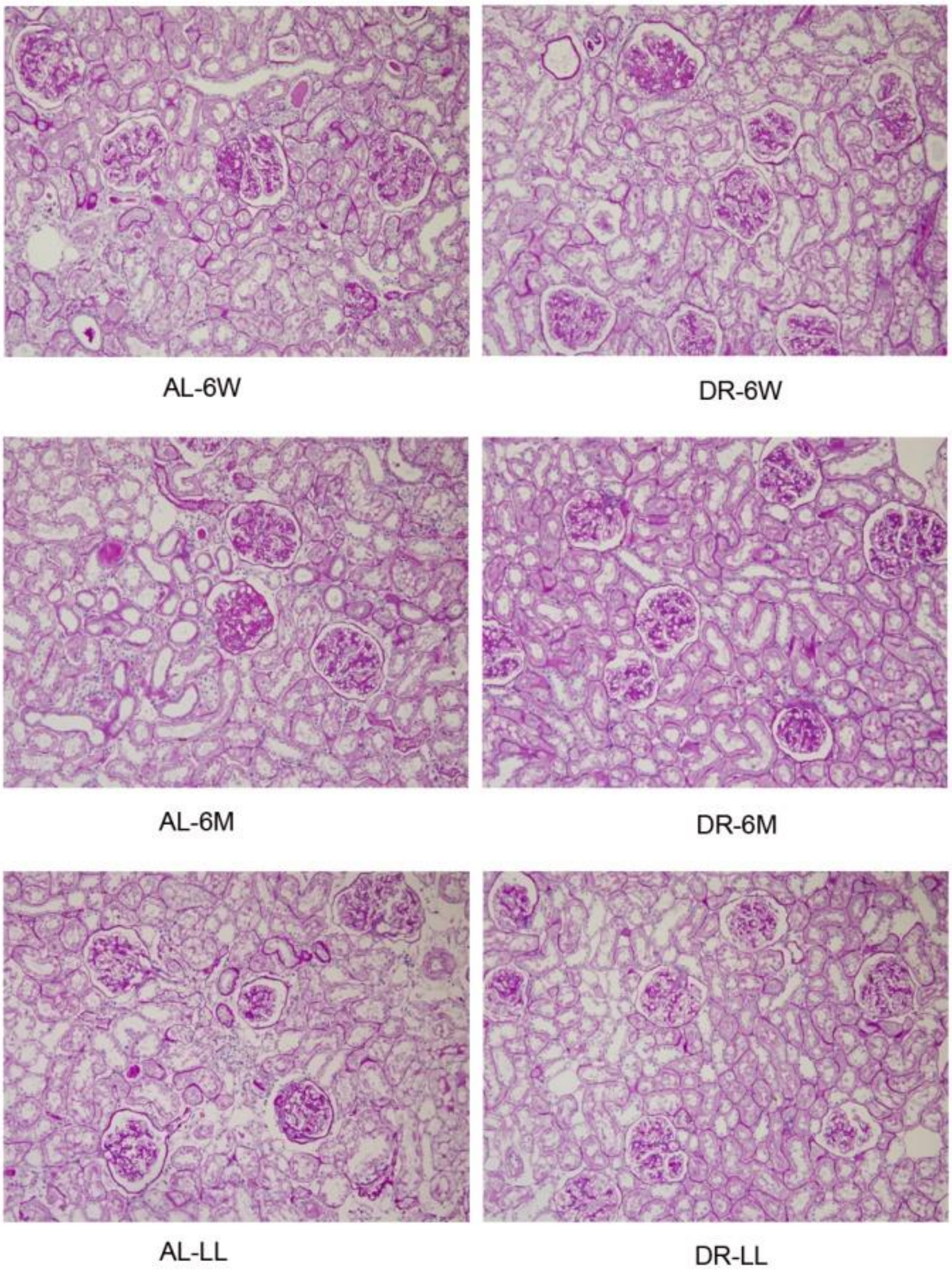
AL-6W
DR-6W
AL-6M
DR-6M
AL-LL
DR-LL

b

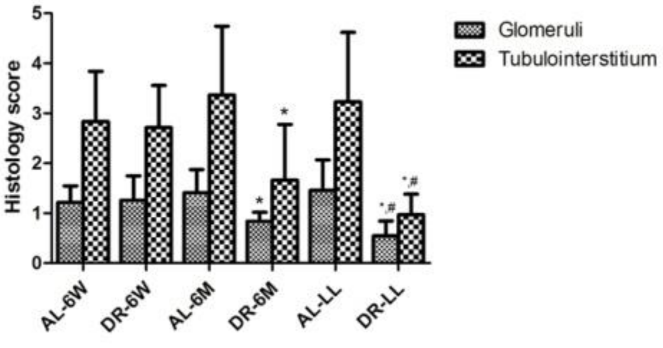
Histology score
0
1
2
3
4
5
*
*
*,#
*,#
AL-6W
DR-6W
AL-6M
DR-6M
AL-LL
DR-LL
Glomeruli
Tubulointerstitium

Fig. S2 Photomicrographs and histology scores of renal cortex in dietary restriction (DR) for different durations. a DR for 6-month (DR-6M) and life-long (DR-LL) alleviated age-related glomerular and tubulointerstitial lesions, but DR for 6-week (DR-6W) couldn't. Renal tissue sections were stained using PAS staining. Magnification, ×200. b Compared with the corresponding AL groups, histology scores were significantly decreased in DR-6M and DR-LL groups rather than in DR-6W. Renal pathological grading by standard procedures from 20 random fields per rat. The data are presented as the mean ± SD (n = 5-8). *$p<0.05$ vs. the corresponding AL. #$p<0.05$ vs. DR-6W.

a

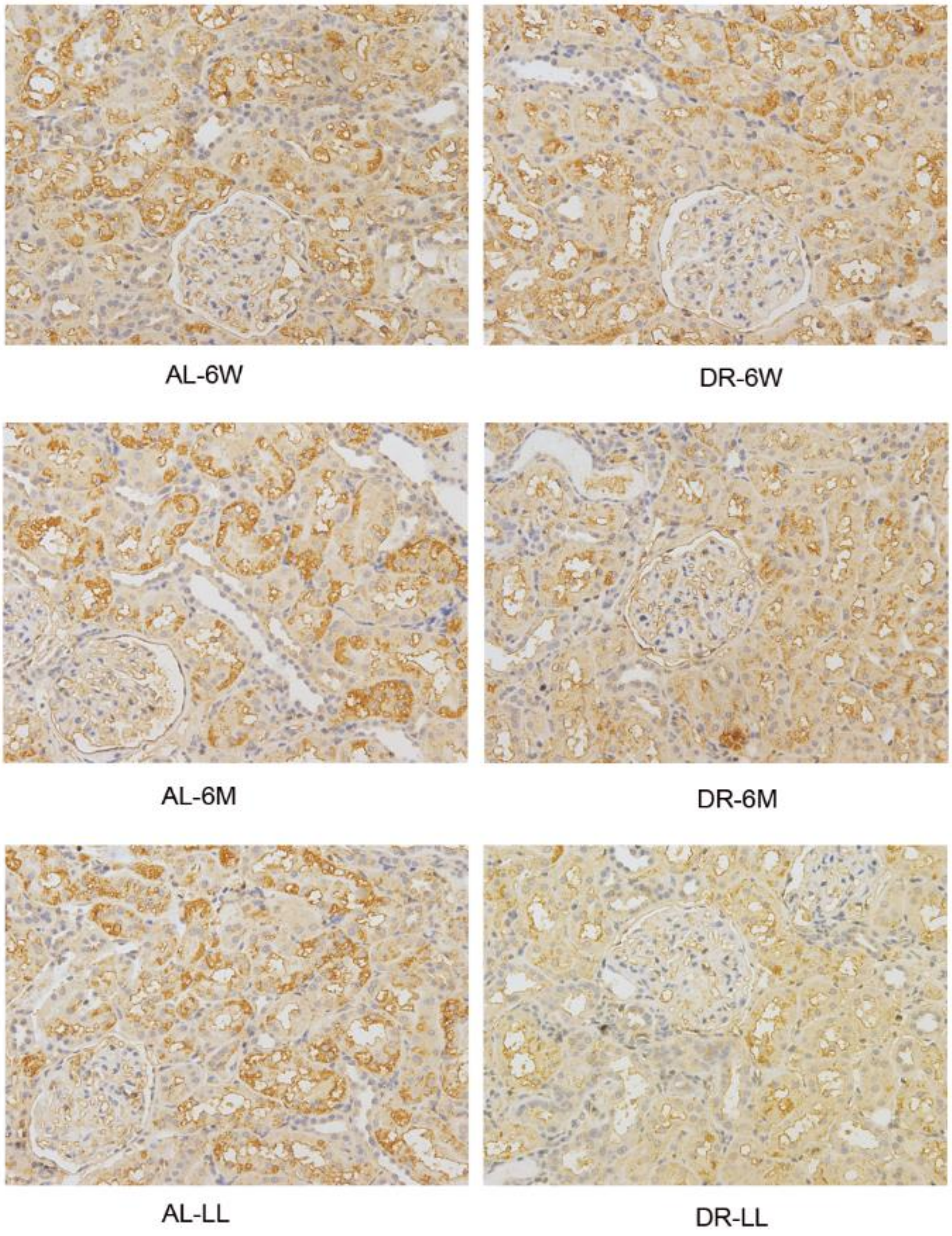
AL-6W
DR-6W
AL-6M
DR-6M
AL-LL
DR-LL

b

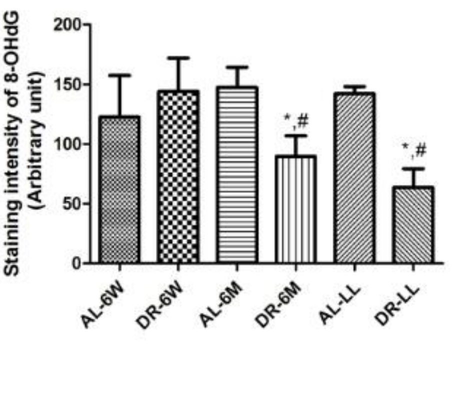
Staining intensity of 8-OHdG
(Arbitrary unit)
200
150
100
50
0
*,#
*,#
AL-6W
DR-6W
AL-6M
DR-6M
AL-LL
DR-LL

Fig.S3 Detection of oxidatively modified DNA in old kidney tissues. a Immunohistochemistry staining results of 8-OHdG in renal tissues, magnification, ×400. It was mainly expressed in the renal tubular cytoplasm.b Quantitative analysis of 8-OHdG immunostaining intensity. Its intensity of immunohistochemical staining was analyzed in ten randomly selected mm^2 areas of renal cortex. The data are presented as the mean ± SD (n = 5-8). *p<0.05 vs. the corresponding AL. #p<0.05 vs. DR-6W.

Table S1 Metabolic indexes and kidney function parameters in the Young-AL and Old-AL groups.

	Young-AL (n=7)	Old-AL (n=19)
Body weight, g	236.3±12.28	684.92±48.69*
Kidney: Body weight, g g-1×100	0.73±0.06	0.57±0.13*
Serum urea nitrogen, mg dL-1	4.32±0.61	6.58±1.13*
Serum creatinine, μmol L-1	29.83±3.13	31.35±5.69
Triglycerides, mg dL-1	0.54±0.10	1.58±1.13*
Total cholesterol,mg dL-1	2.58±0.31	3.34±1.99
Serum glucose, mmol L-1	5.56±0.81	8.52±1.87*
Serum albumin, g L-1	37.5±6.23	34.91±3.23
Total protein, g L-1	62.20±9.31	67.45±4.82
Urine protein/urine creatinine ratio, mg mmol-1	98.69±7.79	450.84±50.72*

Data are mean ± SD

*P < 0.05 vs. Young-AL.

Table S2 Metabolic indexes and kidney function parameters in the aged rats on DR for different durations.

	AL-6W	DR-6W	AL-6M	DR-6M	AL-LL	DR-LL
Body weight, g	683.26±43.12	640.46±71.29	690.88±50.21	595±90.85*	680.63±50.05	540.78±47.91*,#
Kidney: Body weight, g $g^{-1}\times100$	0.59±0.22	0.60±0.16	0.53±0.12	0.62±0.07*	0.58±0.09	0.69±0.1*,#
Serum urea nitrogen, mg dL^{-1}	6.44±1.24	6.00±0.88	6.38±1.27	5.39±0.93*	6.91±0.70	5.16±1.13*
Serum creatinine, μ mol L^{-1}	34.50±9.56	31.51±13.96	30.22±4.97	31.17±2.97	29.32±4.24	34.8±3.94
Triglycerides, mg dL^{-1}	1.33±0.93	1.38±0.95	1.68±0.49	1.02±0.29*	1.72±0.63	0.89±0.24*,#
Total cholesterol,mg dL^{-1}	2.97±2.31	3.39±1.03	3.65±0.83	3.44±0.67	3.41±0.93	2.85±0.70
Serum glucose, mmol L^{-1}	8.54±2.01	7.48±1.78	8.39±0.74	5.02±1.02*,#	8.63±1.01	5.51±0.98*,#
Serum albumin, g L^{-1}	33.91±5.38	33.56±6.12	34.67±3.13	36.05±2.19	36.14±3.37	34.41±3.72
Total protein, g L^{-1}	65.51±6.96	63.5±6.75	67.08±4.13	64.13±3.98	69.77±3.75	66.81±4.15
Urine protein/urine creatinine ratio, mg $mmol^{-1}$	441.8±43.11*	427.44±32.17	439.71±33.07*	362.04±39.69*,#	471.02±48.82	293.70±29.89*,#

Data are mean ± SD (n = 5–8).

*P < 0.05 vs. the corresponding AL.

#P < 0.05 vs. DR-6W.
